# Impact of the COVID‐19 pandemic on Pennsylvania and its healthcare system

**DOI:** 10.1002/hsr2.615

**Published:** 2022-04-22

**Authors:** Ayse Yilmaz, Allison Hermane

**Affiliations:** ^1^ The Hospital and Healthsystem Association of Pennsylvania Harrisburg Pennsylvania USA

**Keywords:** health economics and evaluation, health policy, healthcare management, public health

## Abstract

**Background and Aims:**

Coronavirus disease 2019 (COVID‐19) has taken a toll on Pennsylvania, and the fight against the pandemic continues, with the commonwealth's hospitals and health systems at the epicenter. This report aims to demonstrate the magnitude of the impact of the pandemic on Pennsylvania, with specific attention to its hospitals and health systems and their financial status during the very first year of the pandemic.

**Methods:**

To measure this magnitude, publicly available US and Pennsylvania COVID‐19 data is analyzed, including more detailed geographical, rural/urban, and race/ethnicity analyses.

**Results:**

The results show that the case counts started with just two cases on March 6, 2020, and rose to approximately a million by the first anniversary of the pandemic's arrival in the commonwealth. Tragically, the death toll totaled nearly 25,000 during the first year. Philadelphia County had the highest number of total COVID‐19 cases, while Forest County had the highest incidence rate. The Southeast region had the highest number of total COVID‐19 cases, while the Lehigh Valley had the highest incidence rate. The incidence rate also was higher in rural counties than in urban counties. Black and Latino/a/x populations of Pennsylvania were disproportionally affected by the virus. Several reports measured the financial impact of the pandemic on the state's hospitals to be $4.1–$5 billion during this year.

**Conclusion:**

Hospitals are economic anchors of their communities. To fulfill their critical mission during the pandemic and beyond—and remain economic and community anchors—they need ongoing state and federal support.

## 1 INTRODUCTION

On March 11, 2020, when the World Health Organization (WHO) declared that coronavirus disease 2019 (COVID‐19) was a global pandemic, there were 118,000 confirmed cases in 110 countries.^1^ The first confirmed case in Pennsylvania was recorded on March 6 and the first COVID‐19‐related death occurred on March 18.^2^ Since then, the commonwealth has seen increasing cumulative case counts and deaths. On October 14, after recording more than 1000 new cases each on the previous 9 days, Pennsylvania's then‐Secretary of Health Rachel Levine, MD, announced that the state had entered a second wave.^3^ On December 10, 2020, Pennsylvania saw a single‐day record of 12,816 new cases. By the first anniversary of the pandemic (March 6, 2021), Pennsylvania reported 948,861 cases and 24,425 deaths.^4^

Since the onset of the pandemic, Pennsylvania's hospitals and health systems have heroically stepped up to face the challenges of COVID‐19. To minimize the virus' spread and save lives, the commonwealth's hospitals increased testing efforts, treating, and vaccinating millions of Pennsylvanians. Hospitals erected testing tents, increased intensive care unit (ICU) capacity, and established COVID‐19 units to treat and isolate infected patients while protecting the health of other patients and staff.^5^

These COVID‐19 preparations and precautions came at a great financial cost to hospitals. Revenue shortfalls—due to state and federal government orders requiring hospitals to defer or cancel scheduled services and procedures to free up capacity for COVID‐19 patients—intensified these burdens. In 2020, between increased costs and revenue losses, Pennsylvania hospitals incurred an estimated $5 billion shortfall.^6^

In 2020, hospitals learned a great deal about how to coexist with COVID‐19. They put in place protocols to treat existing COVID‐19 patients, and also safely provide important routine and specialty care. Through reconfiguring space to accommodate for social distancing, creating special wings for COVID‐19 patients, and doubling down on infection prevention and cleaning best practices, hospitals have been able to implement a model for safe care for all who need it.

Hospitals are economic anchors of their communities. Pennsylvania's hospitals and health systems contributed $143 billion in spending including $37 billion in salaries and supported more than 660,000 jobs during the fiscal year (FY) 2019.^7^ Hospitals will need long‐term federal and state support to ensure they can remain economic and healthcare leaders for their communities.

This report aims to summarize how COVID‐19 affected Pennsylvania, with specific attention to its hospitals and health systems.

## 2 COVID‐19 IN NUMBERS

### 2.1 Case history in Pennsylvania

**Figure 1:**
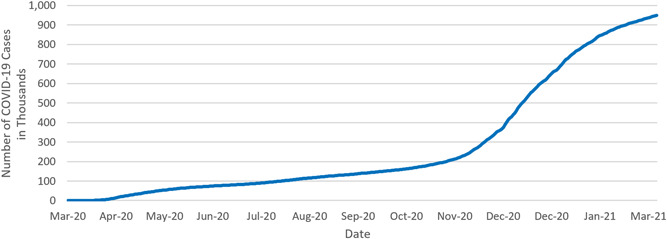
Cumulative COVID‐19 cases in Pennsylvania (in thousands, March 6, 2020–March 6, 2021). COVID‐19, coronavirus disease 2019

Pennsylvania recorded its first confirmed case of COVID‐19 on March 6, 2020, and the total number of cases quickly rose as the testing increased and the virus spread. The rate of increase in COVID‐19 cases slowed during May and June 2020, as compared to April (Figure 1). As the second wave of the pandemic hit Pennsylvania at the beginning of October 2020, the total number of cases started to increase even more dramatically than it did for the first wave. By the end of January 2021, the rate of increase slowed. As of March 6, 2021—exactly 1 year after the first confirmed case in the state—948,861 Pennsylvanians had either confirmed or probable cases of COVID‐19.^4^

**Figure 2:**
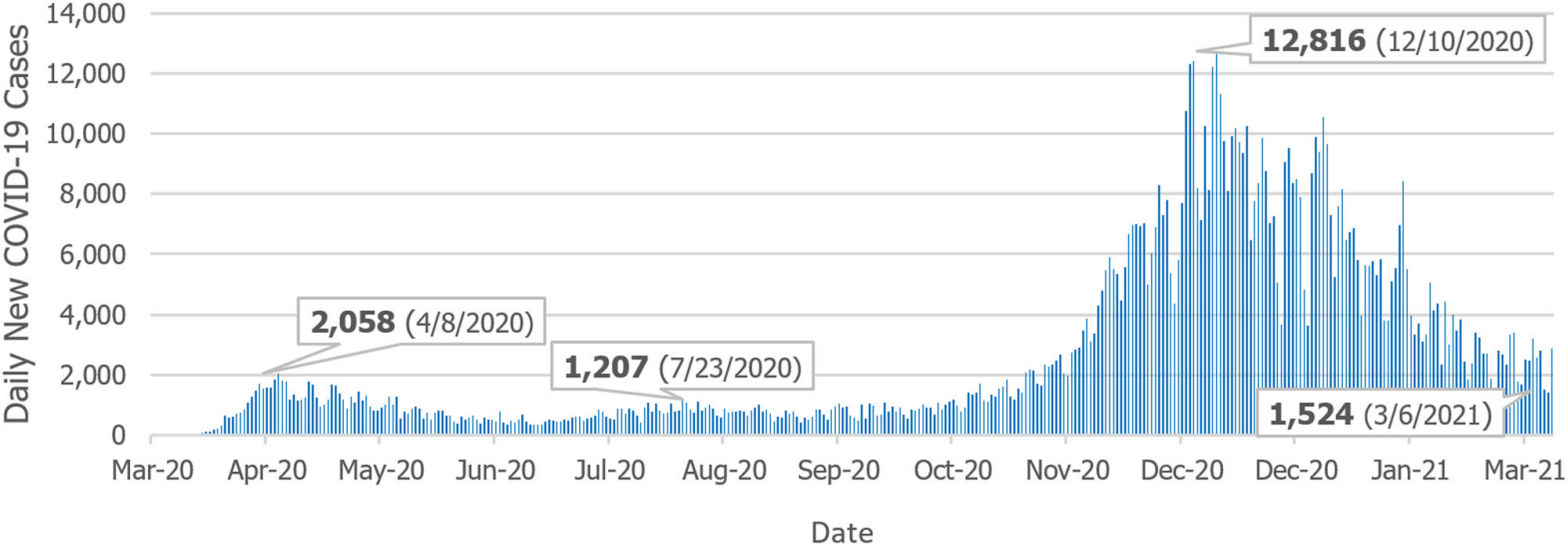
Daily new COVID‐19 cases in Pennsylvania (March 6, 2020–March 6, 2021). COVID‐19, coronavirus disease 2019

As seen in Figure 2, since the onset of COVID‐19, Pennsylvania has seen two notable spikes and one smaller rise in daily case counts. On April 8, 2020, the case count peaked at 2058 cases per day. Another rise in cases occurred during the summer months with a peak of 1207 cases on July 23, 2020. Pennsylvania experienced rapidly increasing case numbers at the start of the second wave. On December 10, 2020, Pennsylvania had the highest daily case count during the pandemic, with 12,816 positives. The daily case counts started to decrease since then, and, by the anniversary of the first recorded case in Pennsylvania (March 6, 2020), there were 1524 daily new cases.^4^

**Figure 3:**
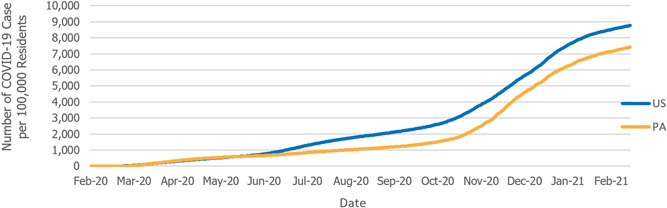
COVID‐19 case rate per 100,000 residents (February 28, 2020–March 6, 2021). COVID‐19, coronavirus disease 2019

The case rate per 100,000 residents for Pennsylvania was on par with the United States until mid‐June when the COVID‐19 case rate was roughly 620 cases per 100,000 (Figure 3). Since that time, the rate of positive COVID‐19 cases for the state of Pennsylvania increased more slowly than the nation as a whole. This coincides with the controlled gradual opening of the Pennsylvania counties which were regulated by the Pennsylvania Department of Health. By early March 2021, the commonwealth had a positive case rate of 7410 per 100,000 residents, while the United States had 8760 positive COVID‐19 cases per 100,000.^8^

### 2.2 COVID‐19 in Pennsylvania's counties and regions

The pandemic followed different trends throughout the commonwealth. This section includes the results of county‐based and regional analyses of the total number of COVID‐19 cases and COVID‐19 incidence rate per 100,000 population.

**Figure 4:**
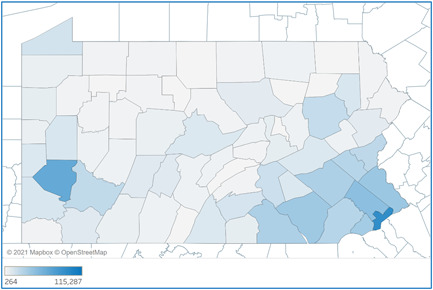
Total COVID‐19 cases by Pennsylvania counties (March 6, 2020–March 6, 2021). COVID‐19, coronavirus disease 2019

During the COVID‐19 pandemic, all 67 counties in Pennsylvania have had residents that tested positive for the coronavirus. Figure 4 shows how the cases were distributed throughout the commonwealth. The darker shaded areas indicate a higher number of total cases, which accumulated around urban areas (Philadelphia and Allegheny counties). Table 1 provides the exact number of total COVID‐19 cases in the top 10 counties with the highest total COVID‐19 cases in Pennsylvania between March 6, 2020, and March 6, 2021. Philadelphia county experienced the highest number of cases (114,350) with nearly 32% more cases compared with the next highest county (Allegheny = 77,994), followed by Montgomery, Bucks, Lancaster, Delaware, York, Berks, Lehigh, and Chester counties.^4^

**Table 1:** Top 10 counties with highest total COVID‐19 cases (March 6, 2020–March 6, 2021)

County	Total COVID‐19 cases
Philadelphia	115,287
Allegheny	78,225
Montgomery	55,200
Bucks	46,151
Lancaster	44,509
Delaware	41,730
York	36,418
Berks	36,369
Lehigh	31,275
Chester	31,218

Abbreviation: COVID‐19, coronavirus disease 2019.

**Figure 5:**
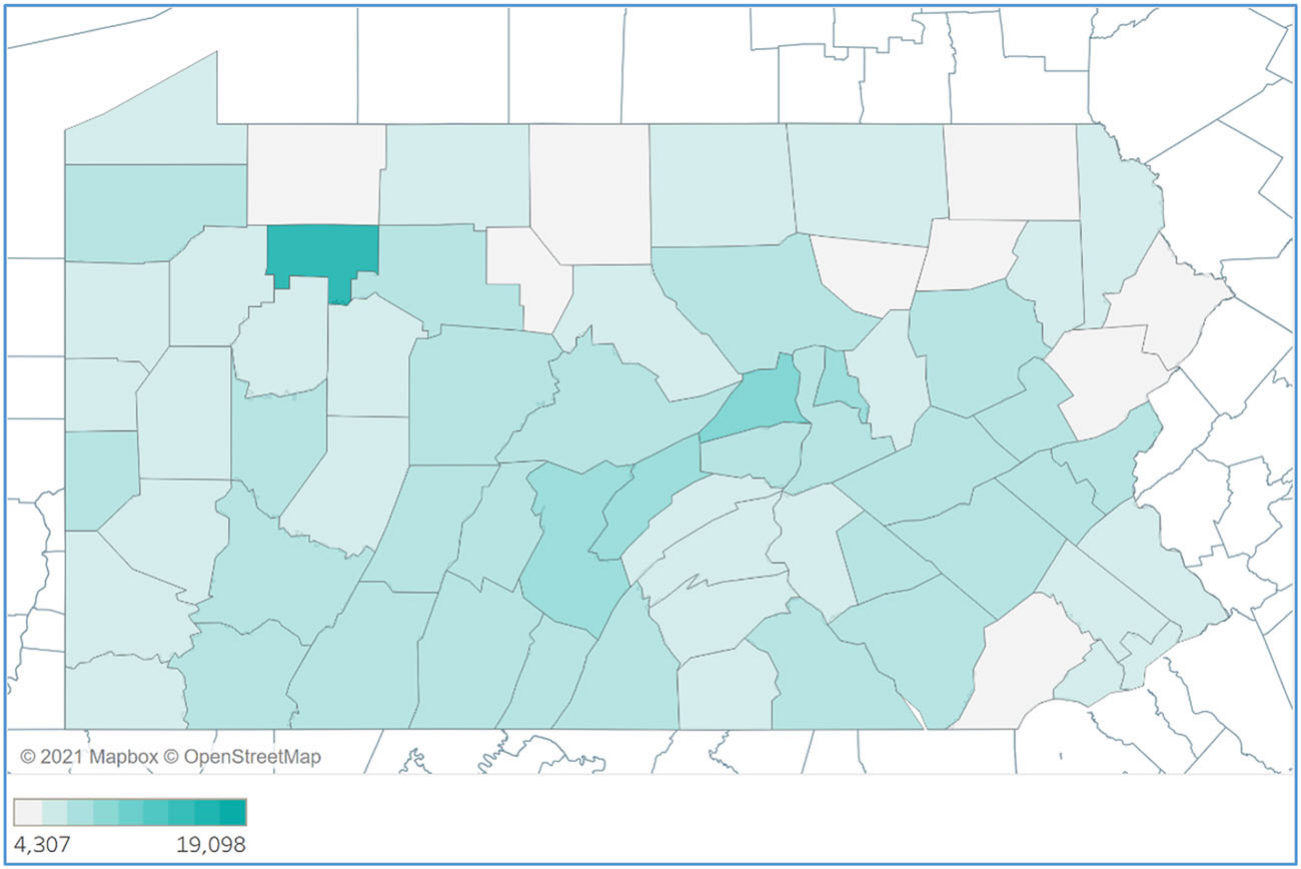
COVID‐19 case rate per 100,000 population by Pennsylvania counties (March 6, 2020–March 6, 2021). COVID‐19, coronavirus disease 2019

Figure 5 demonstrates the rate of COVID‐19 incidence in Pennsylvania counties. The darker shaded areas indicate higher rates. Contrary to the trend of the total number of cases by county, COVID‐19 incidence rates were higher for rural counties. Table 2 lists the top 10 Pennsylvania counties with the highest COVID‐19 case rate per 100,000 residents between March 6, 2020, and March 6, 2021. Forest County observed the highest rate with 19,098 COVID‐19 cases per 100,000 population. Eight of the top 10 counties with the highest incidence rates are rural counties (Lebanon and Northampton counties were the exceptions). This is in line with an overall higher rate of incidence in rural counties of Pennsylvania, compared to urban counties (Figure 8).^4^

**Table 2:** Top 10 counties with highest COVID‐19 case rate per 100,000 residents (March 6, 2020–March 6, 2021)

County	Rate
Forest	19,098
Union	11,756
Mifflin	10,029
Montour	9627
Huntingdon	9556
Lebanon	9180
Somerset	9152
Cambria	9075
Northampton	8993
Northumberland	8907

Abbreviation: COVID‐19, coronavirus disease 2019.

**Figure 6:**
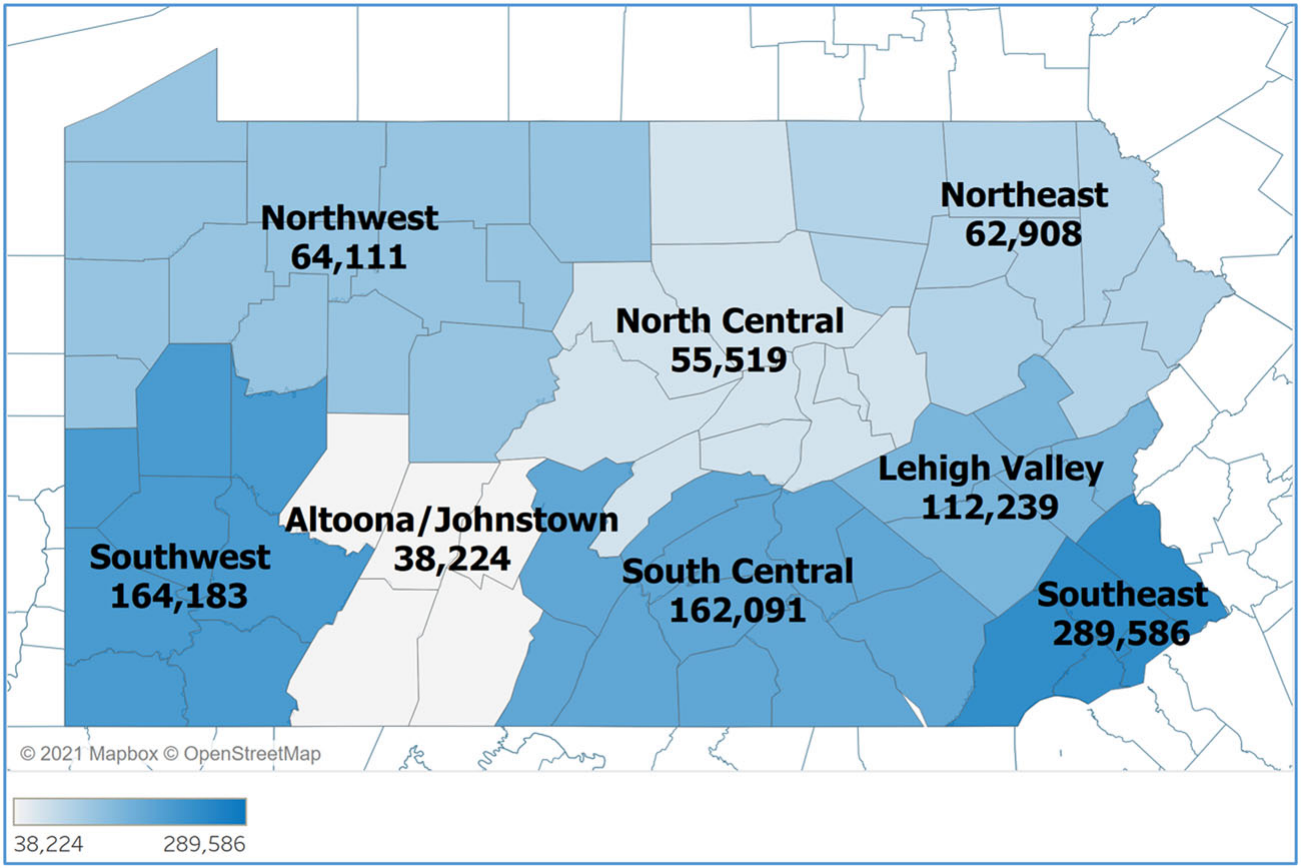
Total cases by Pennsylvania regions (March 6, 2020–March 6, 2021).

Figure 6 lays out the distribution of the cumulative COVID‐19 cases in Pennsylvania by its eight regions. Of the total 948,861 COVID‐19 cases in Pennsylvania between March 6, 2020, and March 6, 2021, the Southeast region had the highest number of cases (darkest shaded area, with 289,586 cases). This was followed by the Southwest, South Central, Lehigh Valley, Northwest, Northeast, North Central, and Altoona/Johnstown regions, in the order of decreasing total case numbers*.^4^

**Figure 7:**
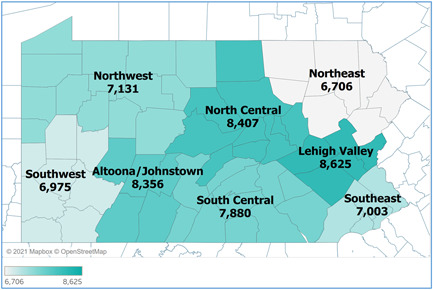
COVID‐19 Case Rate per 100,000 Residents Pennsylvania Regions (March 6 2020‐March 6, 2021)

Figure 7 demonstrates the COVID‐19 incidence rates of Pennsylvania's eight regions. Lehigh Valley, North Central, and Altoona/Johnstown regions (darker shaded areas) had incidence rates that are higher than 8000 cases/100,000 population, which are followed by South Central, Northwest, and Southeast regions with incidence rates between 7000 and 8000 cases/100,000 population. Southwest and Northeast regions had the lowest rates that are smaller than 7000 cases/100,000 population.^4^

### 2.3 COVID‐19 incidence in rural and urban Pennsylvania

According to the Centers for Disease Control and Prevention's (CDC) analysis of COVID‐19 incidence by urban‐rural classification, nationally, large central and large fringe metropolitan areas had the highest COVID‐19 incidence early in the pandemic (mid‐March to mid‐May). However, since September 2020, COVID‐19 started spreading into rural communities faster, resulting in the highest incidence in medium/small metropolitan areas and micropolitan/noncore areas until November 2020^†^.^9^

**Figure 8:**
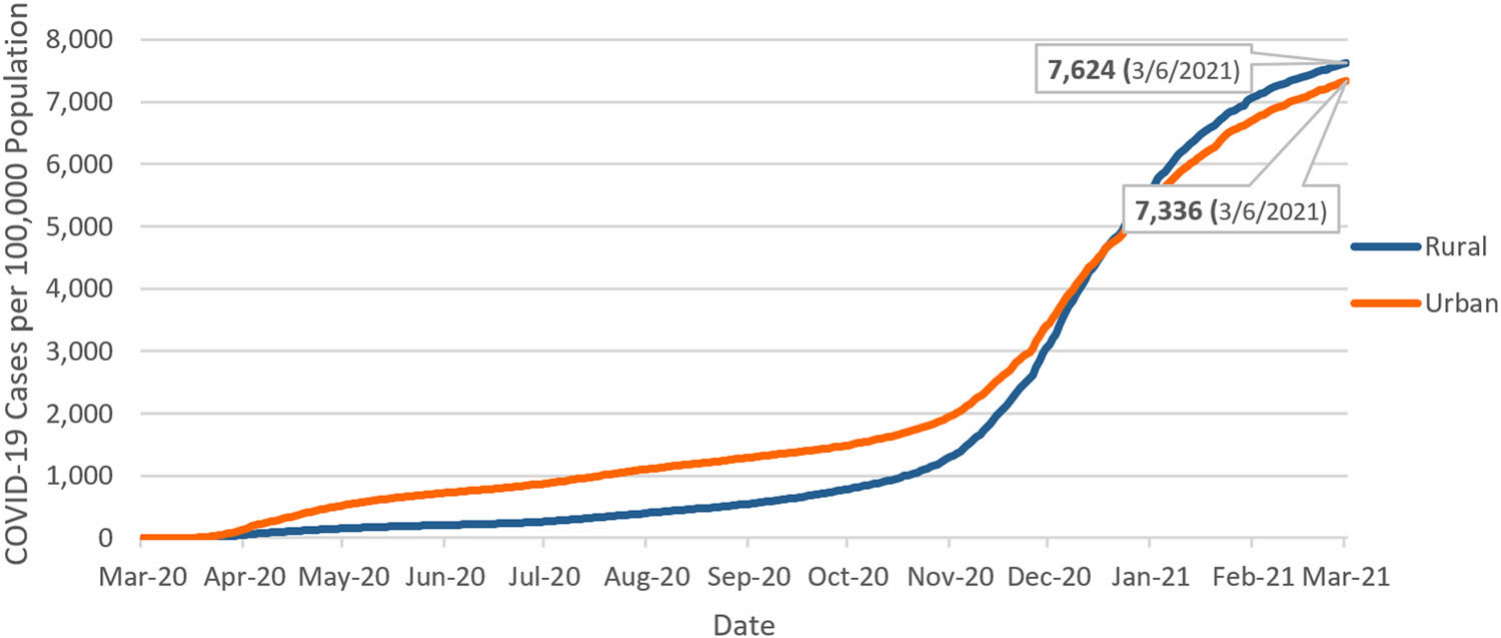
Cumulative case rate: Urban versus rural Pennsylvania (March 6, 2020–March 6, 2021).

The rural population consists of 26% of Pennsylvania's total population, which makes the commonwealth the state with the third‐largest rural population.^10^, ^11^ When we look at this crucial piece of Pennsylvania's overall population, rural counties in Pennsylvania had a higher overall incidence rate than its urban counties based on the number of total cases between March 6, 2020, and March 6, 2021 (Figure 8). The trend observed in Pennsylvania was similar to the nationwide trend of rural communities having higher overall rates during the later phases of the pandemic.

### 2.4 Hospitalizations due to COVID‐19 in Pennsylvania

**Figure 9:**
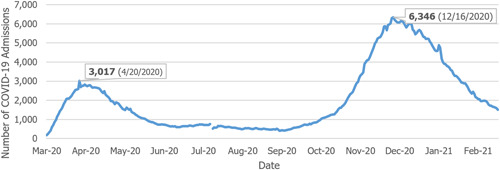
Hospitalized COVID‐19 patients in Pennsylvania (March 26, 2020–March 6, 2021). COVID‐19, coronavirus disease 2019

Figure 9 shows that COVID‐19‐related hospitalizations have increased in proportion to the number of positive cases reported. According to the data made available by the Pennsylvania Department of Health, the highest number of hospitalizations at the beginning stages of the pandemic was 3017, recorded on April 20, 2020. The most significant peak in COVID‐19‐related hospitalizations in Pennsylvania occurred on December 16, 2020, with 6346 COVID‐19‐related hospitalizations. The missing data and change in the data on July 30, 2020, is due to a change in the criteria for reporting COVID‐19‐related deaths^§^.^12^, 3

### 2.5 Rate of COVID‐19 patients to other inpatients

**Figure 10:**
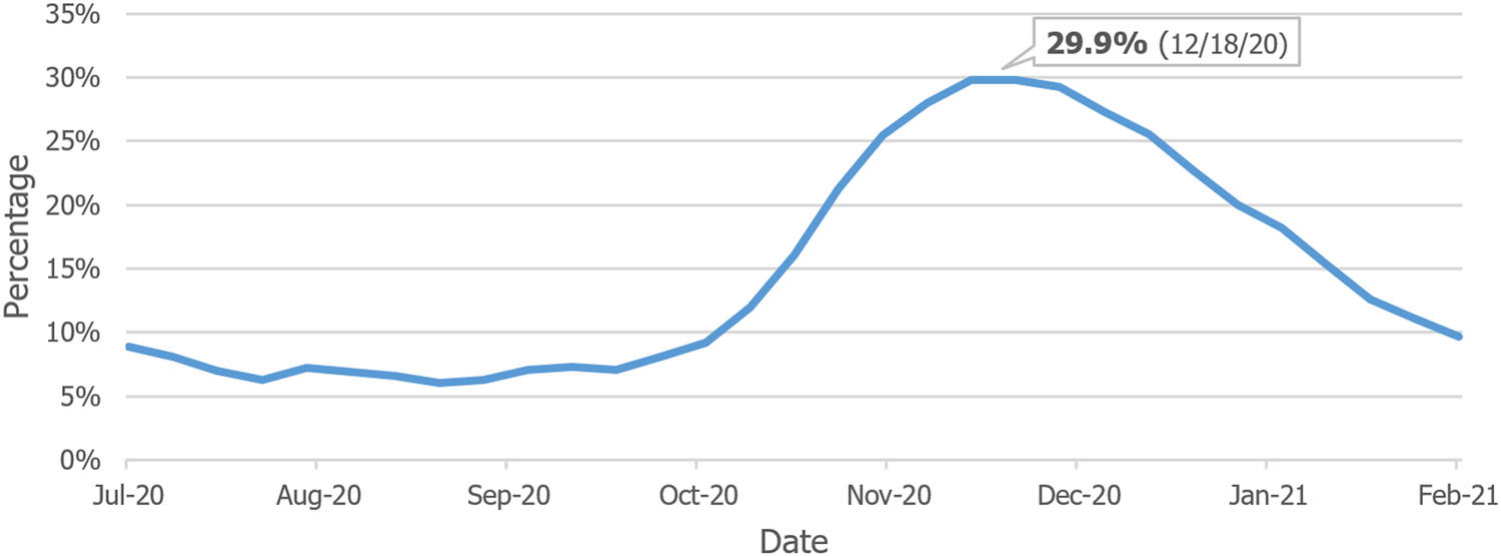
Percentage of COVID‐19 patients to all inpatients (July 31, 2020–February 26, 2021). COVID‐19, coronavirus disease 2019

As seen in Figure 10, at the end of July 2020, 7.5% of all adult inpatients were hospitalized for COVID‐19 (based on the sum of confirmed and suspected COVID‐19 hospitalizations). This number peaked at 29.9% on December 18, 2020.^13^

As the rates of patients who needed acute hospital care increased, hospitals had to address several challenges, including addressing the need for more clinical staff and sourcing critical supplies—such as hospital beds, personal protective equipment, medicines, and ventilators.^14^ For example, The Center for Infectious Disease Research and Policy reported that, nationwide, hospitals had been struggling with COVID‐19‐related nursing shortages.^15^

### 2.6 Death rate due to COVID‐19 in Pennsylvania

**Figure 11:**
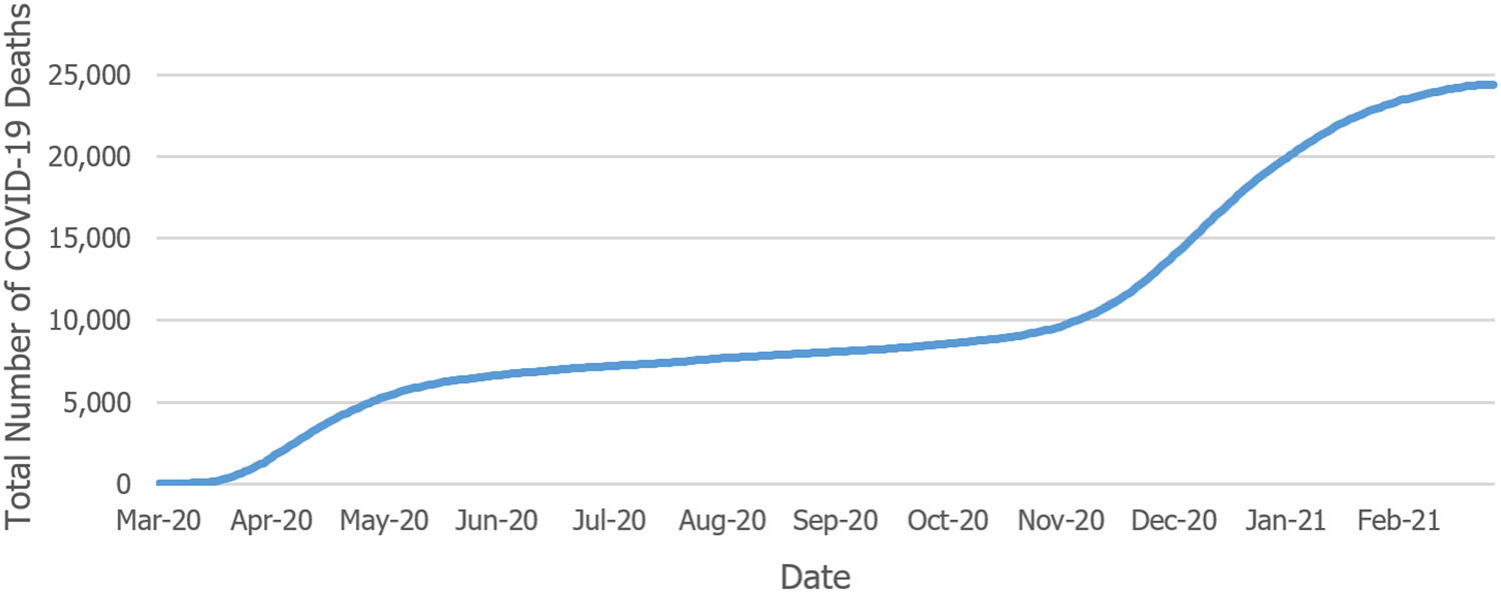
Cumulative deaths due to COVID‐19 in Pennsylvania.

Figure 11 illustrates the trend of COVID‐19‐related deaths in Pennsylvania. The number of deaths due to COVID‐19 increased as the number of those infected was on the rise. The total number of COVID‐19‐related deaths in the state of Pennsylvania was 24,425 as of March 6, 2021¶.^16^, 4

**Figure 12:**
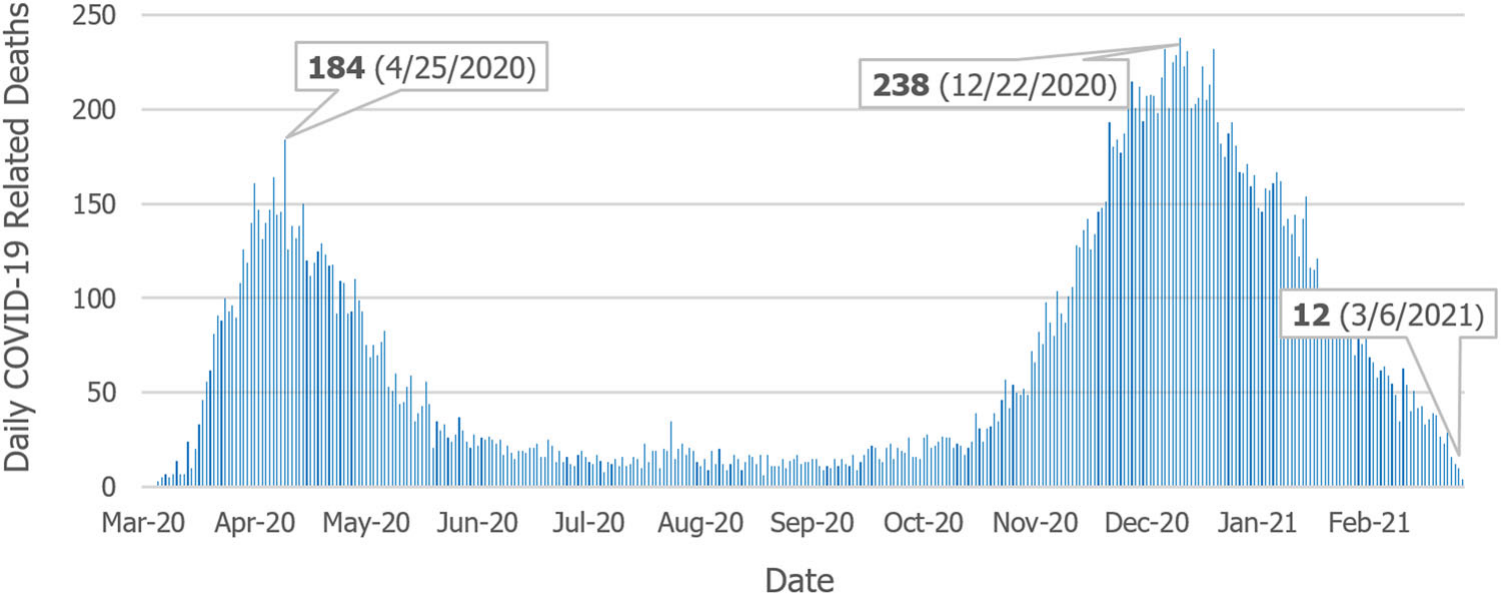
New deaths per day (March 18, 2020–March 8, 2021).

As cases began to rise in the state, the deaths also increased. As seen in Figure 12, during the first spike in COVID‐19 cases, the worst day was April 25, 2020, with 184 deaths. The daily recorded deaths due to COVID‐19 remained consistent between the first and the second waves but started to increase dramatically by the end of October 2020. The second wave peaked with the most single‐day deaths reaching 238 on December 22, 2020. On March 6, 2020, Pennsylvania experienced 12 COVID‐19‐related deaths.^16^

**Figure 13:**
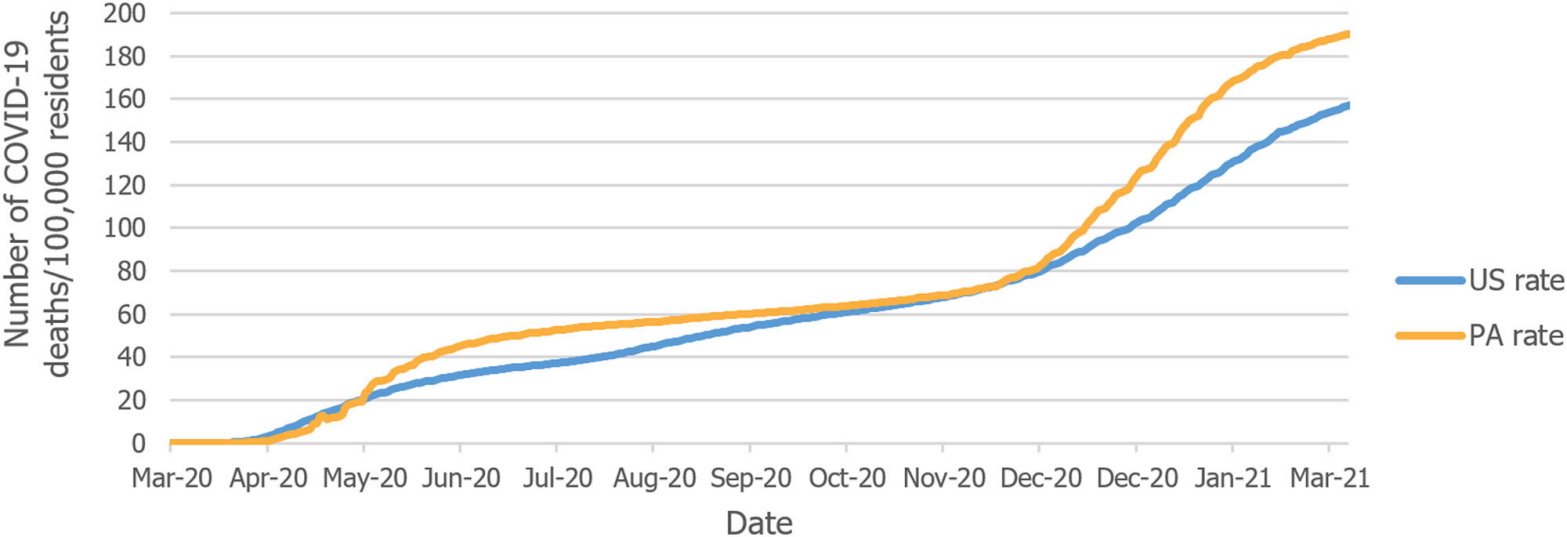
The death rate per 100,000 residents in Pennsylvania and United States.

Figure 13 compares the rate of COVID‐19‐related deaths per 100,000 residents in the United States and Pennsylvania. Overall, the COVID‐19‐related death rate for the commonwealth is higher than it is nationwide, which contradicts an overall lower rate of COVID‐19 cases Pennsylvania experienced compared to the United States (Figure 3). This may be attributed to a higher rate of people who are 65 years or older, relative to the general population in Pennsylvania (18.7% in Pennsylvania, compared to 16.5% nationwide).^8^, ^17^

### 2.7 Differences in the incidence of COVID‐19 for racial and ethnic groups in Pennsylvania

**Figure 14:**
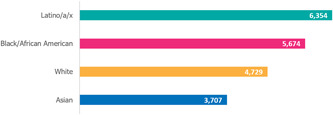
Cases per 100,000 people in Pennsylvania.

**Figure 15:**
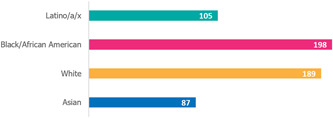
Deaths per 100,000 people in Pennsylvania.

Data suggests that COVID‐19 has disproportionately affected minority racial and ethnic groups in the commonwealth. Figures 14 and 15 show that the Hispanic/Latino population and the Black/African American population have higher rates of COVID‐19 cases than the white population. The figure also illustrates how the mortality rate for the Black/African American population is higher than any other racial or ethnic group^#,$^.^18^

## 3 POLICY AND REGULATIONS PROMOTING MITIGATION^19^

**Figure 16:**
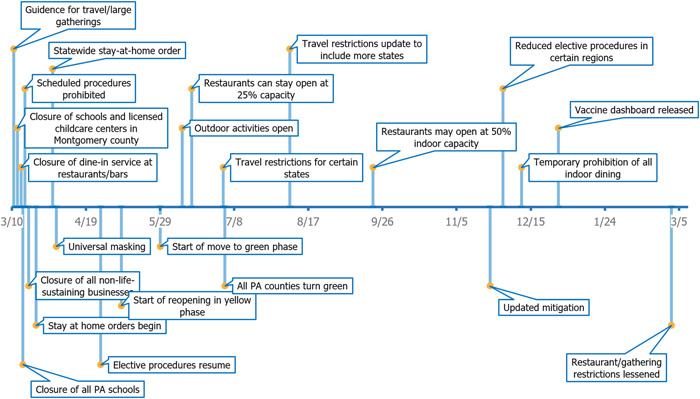
Timeline of major mitigation efforts put into effect in Pennsylvania (March 11, 2020–March 6, 2021).

In consultation with the Pennsylvania Department of Health, the state has been updating the mitigation efforts based on the spread of the pandemic. Below is a timeline (Figure 16) and a table (Table 3) of mitigations that went into effect between March 11, 2020, and March 6, 2021.

**Table 3:** Mitigation efforts were put into effect in Pennsylvania (Mar 11, 2020–March 6, 2021)

Date	Regulations
11/3/2020	Guidance for travel/large gatherings
13/3/2020	Closure of schools and licensed childcare centers in Montgomery County
15/3/2020	Closure of dine‐in service at restaurants/bars
16/3/2020	Closure of all Pennsylvania schools
17/3/2020	Suspension of scheduled procedures
19/3/2020	Closure of all nonlife‐sustaining businesses
23/3/2020	Stay‐at‐home orders begin
1/4/2020	Statewide stay‐at‐home order
3/4/2020	Universal masking
27/4/2020	Resumption of scheduled procedures
8/5/2020	Start of reopening in yellow phase**
29/5/2020	Start of the move to green phase
10/6/2020	Outdoor activities open
15/6/2020	Restaurants can stay open at 25% capacity
2/7/2020	Travel restrictions for certain states
3/7/2020	All Pennsylvania counties turn green
7/8/2020	Travel restrictions updated to include more states
21/9/2020	Restaurants may open at 50% indoor capacity
23/11/2020	Updated mitigation
30/11/2020	Reduction of scheduled procedures in certain regions
10/12/2020	Temporary prohibition of all indoor dining
30/12/2020	COVID‐19 vaccine dashboard initiated^‡^
1/3/2021	Changes to maximum occupancy limits and elimination of out‐of‐state travel restrictions

## 4 FINANCIAL TOLL OF COVID‐19 FOR PENNSYLVANIA HOSPITALS

Since its onset, hospitals have been at the center of the fight against the COVID‐19 pandemic. Health care providers put their lives on the line to treat severely ill patients, and hospitals faced tremendous economic burden—not just because of limited operability mandated by the government and public anxiety towards seeking primary care, but also resource‐intensive COVID‐19 admissions.

### 4.1 Analysis of the impacts of COVID‐19 on Pennsylvania Hospitals^6^

In August 2020, Health Management Associates (HMA) interviewed leaders of 12 Pennsylvania health systems to assess COVID‐19's current and future impacts on the commonwealth's hospitals. HMA's analysis of this data revealed that, due to the pandemic, hospitals experienced plummeting patient volumes, large margin shortfalls, and many other challenges.

#### 4.1.1 Hospitals did not expect to reach prepandemic patient volume during 2021

Early during the pandemic—facing a novel virus with unknown impacts—state and federal governments issued orders for hospitals to cancel or defer all scheduled/nonemergency services to prepare for a major surge of COVID‐19 patients. During May, the commonwealth allowed a phased approach to bring scheduled services back online, and some patient volumes increased; however, all interviewed health systems reported that they did not expect to reach prepandemic activity levels during 2020.

For the second half of the year, hospitals anticipated the remaining 5% below historical levels in inpatient discharges and 13% below pre‐pandemic experience in emergency room visits and surgeries.

#### 4.1.2 Federal relief fell short in covering hospitals' financial losses

From mid‐March to July 2020, Pennsylvania hospitals experienced an estimated $5 billion shortfall in operating margins which represents a 24% drop from pre‐pandemic levels. The estimated $2.8 billion federal relief received so far falls short in covering the financial loss of Pennsylvania hospitals due to COVID‐19.

Revenue losses and increased expenses dramatically changed the fiscal landscape and long‐term viability of hospitals across the state.

According to the Pennsylvania Health Care Cost Containment Council (PHC4), 34% of Pennsylvania's hospitals operated with a negative margin in 2019. These hospitals entered the pandemic with relatively weak balance sheets, and COVID‐19‐related margin shortfalls threaten their ability to serve their communities.

#### 4.1.3 COVID‐19 contributed to the other challenges hospitals are facing

In addition to the impacts on public health and the hospital community's financial stability, the COVID‐19 pandemic has had notable impacts on several aspects of the state's economy and healthcare system.

The pandemic has changed dynamics within the healthcare workforce, due to healthcare workers being unable to work because of illness or exposure to COVID‐19, deciding to retire early, taking leaves of absence out of increased health risk concerns, facing the stress and emotional strain of the pandemic, or experiencing difficulty securing child care.

The commonwealth experienced increased unemployment since the start of the pandemic. The 4.7% unemployment rate during February 2020 peaked at a rate of 16.1% during April 2020. Even though many people were able to go back to work, as of November 2020 Pennsylvania's unemployment still was higher than the prepandemic economic environment with a rate of 6.6%.^20^

People lost their health coverage as they lost their jobs. Another report by HMA estimated that close to one million Pennsylvanians could lose employer‐sponsored health insurance coverage as a result of the pandemic.^21^ This will negatively impact hospitals through changes in payor mix and increasing uncompensated care (care that hospitals provide, but for which they do not receive payment from private or commercial insurance providers; this includes charity care). Even before the onset of COVID‐19, Pennsylvania hospitals and health systems provided a vast amount of uncompensated care. According to PHC4, uncompensated care for Pennsylvania general acute hospitals increased from $750 million during FY 2018, to $820 million during FY 2019—an increase of 9.3%. This represented the first year‐to‐year increase in uncompensated care during the past 5 years.^22^

### 4.2 COVID‐19 disaster emergency report^23^

PHC4 also performed a study to investigate the financial impact of COVID‐19 on Pennsylvania hospitals. The study—which was based on the information provided by 91% of hospitals across the state—confirms the enormous damaging impact that the COVID‐19 pandemic has had on the financial health of hospitals through the first three quarters of 2020. According to the study, Pennsylvania hospitals:

- Experienced revenue losses of $4.1 billion, due to the suspension of scheduled/non‐emergent services and decreases in nonscheduled/emergent services not related to COVID‐19.
- Incurred $81 million in costs related to COVID‐19 testing, including costs related to commercial lab services.
- Expended $349 million for increased staffing and labor costs to expand services and staff emergency operations centers.
- Devoted $258 million to purchase additional supplies and equipment, such as personal protective equipment, computer hardware, and temporary tents.
- Incurred $21 million in costs to set up emergency operation centers, including construction and retrofitting facilities to provide separate screening and security areas.
- Expended $89 million for other miscellaneous activities such as providing housing and care for patients who do not require hospitalization, obtaining consulting services to comply with COVID‐19‐related operations, and other expenses to prevent, prepare, and respond to the pandemic.

This report complements the analysis commissioned by HAP and prepared by HMA, which outlined the significant financial consequences on the hospital community through the end of July 2020.

### 4.3 COVID‐19 in 2021: The potential effect on the hospital revenues^24^

Kaufman Hall—an independent healthcare management consulting organization—analyzed historical hospital revenues and possible paths of hospital volumes, vaccine progress, and decline in COVID‐19 cases to forecast 2021 hospital revenue. Both of the optimistic and pessimistic scenarios presented in the report show a significant revenue loss compared with what would be expected without the effect of COVID‐19.

The report's key findings include:

- Under an optimistic scenario, US hospitals could face a total revenue loss of $53 billion during 2021—including $27 billion in outpatient revenue, $17 billion in inpatient revenue, and $9 billion in emergency department revenue.
- Under a pessimistic scenario, US hospitals could face a total revenue loss of $122 billion during 2021—including $64 billion in outpatient revenue, $41 billion in inpatient revenue, and $17 billion in emergency department revenue.
- Hospitals experienced increased expenses, in addition to revenue losses. During 2020, hospitals reported a 17% increase in drug expenses, a 16% increase in purchased service expenses, a 14% increase in labor expenses, and a 13% increase in supply expenses—all of which could continue into 2021 as the pandemic continues.

## 5 ECONOMIC IMPACT OF PENNSYLVANIA HOSPITALS^7^

Hospitals are economic anchors in their communities. Hospitals directly impact their communities' economies in many ways—maintaining and constructing new buildings; providing jobs; and purchasing medical supplies, pharmaceuticals, and medical equipment. Hospitals also indirectly impact the economy through business interactions with organizations from other industries, such as employment and cleaning services. Finally, hospitals induce economic activity outside of the hospital in such industries as real estate, financial investment firms, and restaurants. They also attract federal research dollars to the state, enabling Pennsylvania to develop innovations that improve care for all Americans.

During 2019, Pennsylvania's hospitals provided the commonwealth with a total economic value of $143 billion in spending. This includes:

- $64 billion in direct impact, representing the dollars hospitals payout for employee salaries, wages, and benefits, as well as for the many goods and services needed to provide healthcare services and support hospitals and health system operations.
- $79 billion in ripple impacts that represent the additional economic activity that results from the circulation of hospital dollars in local communities and across the state.

The total economic value includes $37 billion in salaries, which support thousands of Pennsylvania families. On their own, Pennsylvania's hospitals, directly and indirectly, supported more than 660,000 jobs during 2019, accounting for approximately one of every nine jobs in the state. This includes:

- Directly employing more than 291,000 Pennsylvanians in a wide variety of jobs, providing nearly $19.2 billion in wages, salaries, and benefits.
- Supporting more than 370,000 additional jobs—that pay another $18 billion in salaries—through the direct purchase of goods and services and the subsequent circulation of hospital dollars in local economies.

Helping to pave the way for new evidence‐based technology and cutting‐edge care delivery, during 2019 alone, Pennsylvania's hospitals and universities with hospital‐affiliated medical schools attracted an estimated $1.85 billion in federal funds. These investments are designed to improve health and healthcare delivery not just for Pennsylvanians, but for patients across the country and around the world.

In addition, commonwealth hospitals serve their communities through educating tomorrow's healthcare professionals, providing both charity care and unreimbursed care, and increasing the productivity of Pennsylvania's workforce.

## 6 CONCLUSION

COVID‐19 has taken a toll on Pennsylvania, and the fight against the pandemic continues, with the commonwealth's hospitals and health systems at the epicenter. With just two cases on March 6, 2020, the case counts rose to approximately a million by the first anniversary of the pandemic's arrival in the commonwealth. Tragically, the death toll totaled nearly 25,000 during the first year.^4^, ^16^

Pennsylvania appears to have contained the virus more successfully when compared to the rest of the nation. The state has employed strong mitigation efforts throughout the pandemic and healthcare providers have worked hard to treat Pennsylvania patients.^8^, ^19^ The pandemic followed different trends throughout the state. Philadelphia County had the highest number of total COVID‐19 cases among all Pennsylvania counties, while Forest County had the highest incidence rate per 100,000 populations. The Southeast region had the highest number of total COVID‐19 cases, while the Lehigh Valley had the highest incidence rate per 100,000 populations. The incidence rate also was higher in rural counties than in urban counties, based on the total number of cases between March 6, 2020, and March 6, 2021.^4^ Black and Latino/a/x populations of Pennsylvania were disproportionally affected by the virus.^18^

The COVID‐19 pandemic also threatened the financial stability of hospitals and the economic foundation that the commonwealth's hospitals and health systems provide. According to an HMA report, Pennsylvania's hospitals and health systems experienced an estimated loss of more than $5 billion due to temporary closures and curtailing non‐emergent treatment.

Other reports also confirmed COVID‐19's financial impact on Pennsylvania's hospitals and affirmed the need for their long‐term financial stability. PHC4 published a report based on the actual data of most of the Pennsylvania hospitals and measured the revenue loss of the hospital community as $4.1 billion through the first three‐quarters of 2020.^23^ Kauffman Hall estimated the 2021 revenue loss of US hospitals to be between $53 and $122 billion. The report suggests that the COVID‐19‐related expenses hospitals had to incur during 2020 may continue during 2021, in addition to these revenue losses.^24^

The estimated $2.8 billion federal relief received so far falls short in covering the financial loss.^6^ A subsequent HMA report estimated that one million Pennsylvanians could lose employer‐sponsored health insurance coverage as a result of the pandemic, which will negatively impact hospitals through changes in payor mix and increasing uncompensated care.^21^

Hospitals are economic anchors of their communities. During FY 2019, Pennsylvania's hospitals and health systems contributed $143 billion to state and local economies. This figure includes $37 billion in salaries which support thousands of Pennsylvania families. The hospital community also supported more than 660,000 jobs and $1.85 billion of federal research funds.^7^

For hospitals to fulfill their critical mission during the pandemic and beyond—and remain economic and community anchors—they need ongoing state and federal support. In 2021, hospitals will need these resources as they deliver outstanding care and administer the COVID‐19 vaccine to patients across the commonwealth.

With the identification of the disparities and challenges COVID has presented before us, efforts and resources could be more efficiently targeted and focused on addressing them. Moving forward, we encourage improvements in health information exchange (HIE) to improve coordinated and timely care between different healthcare providers, public health, and other stakeholders.

## AUTHOR CONTRIBUTIONS

*Conceptualization*: Ayse Yilmaz. *Formal Analysis*: Ayse Yilmaz. *Investigation*: Ayse Yilmaz and Allison Hermane. *Methodology*: Ayse Yilmaz. *Visualization*: Ayse Yilmaz. *Writing—Original Draft Preparation*: Ayse Yilmaz and Allison Hermane. *Writing—Review & Editing*: Ayse Yilmaz and Allison Hermane. All authors have read and agreed to the final version of the manuscript. Ayse Yilmaz has full access to all of the data in this study and takes complete responsibility for the integrity of the data and the accuracy of the data analysis.

## CONFLICTS OF INTEREST

The authors declare no conflicts of interest.

## TRANSPARENCY STATEMENT

Ayse Yilmaz affirm that this manuscript is an honest, accurate, and transparent account of the study being reported; that no important aspects of the study have been omitted; and that any discrepancies from the study as planned (and, if relevant, registered) have been explained.

## Data Availability

The authors confirm that the data supporting the findings of this study are available within the article [and/or] its supplementary materials.

## ENDNOTES

3 The reporting requirements, hence the hospitalization numbers, changed July 30 when hospitals started reporting to Department of Health and Human Services (HHS). Previously, hospitals reported patient data to the Centers for Disease Control and Prevention and Pennsylvania government using a specific database.

4 According to COVID Tracking Project, On April 23, 2020, Pennsylvania revised down its total deaths (confirmed and probable) from 1,622 to 1,421 to exclude some probable deaths that needed further confirmation to be linked to COVID‐19.
